# Manufacturing Process of Polymeric Microneedle Sensors for Mass Production

**DOI:** 10.3390/mi12111364

**Published:** 2021-11-05

**Authors:** Jae Yun Baek, Kyung Mook Kang, Hyeong Jun Kim, Ju Hyeon Kim, Ju Hwan Lee, Gilyong Shin, Jei Gyeong Jeon, Junho Lee, Yusu Han, Byeong Jun So, Tae June Kang

**Affiliations:** Advanced Materials Lab, Department of Mechanical Engineering, Inha University, Incheon 22212, Korea; jaeyun.baek@inha.edu (J.Y.B.); athrun93@naver.com (K.M.K.); hyeongjun2531@gmail.com (H.J.K.); juhyun4280@gmail.com (J.H.K.); Juhwanlee3260@gmail.com (J.H.L.); mysky24sky@gmail.com (G.S.); newjg91@nate.com (J.G.J.); lmy2415@gmail.com (J.L.); yousoo0519@naver.com (Y.H.); bangjun0314@gmail.com (B.J.S.)

**Keywords:** microneedle, laser machining, polylactic acid, electrochemical detection, biomolecules

## Abstract

In this work, we present a fabrication process for microneedle sensors made of polylactic acid (PLA), which can be utilized for the electrochemical detection of various biomarkers in interstitial fluid. Microneedles were fabricated by the thermal compression molding of PLA into a laser machined polytetrafluoroethylene (PTFE) mold. Sensor fabrication was completed by forming working, counter, and reference electrodes on each sensor surface by Au sputtering through a stencil mask, followed by laser dicing to separate individual sensors from the substrate. The devised series of processes was designed to be suitable for mass production, where multiple microneedle sensors can be produced at once on a 4-inch wafer. The operational stability of the fabricated sensors was confirmed by linear sweep voltammetry and cyclic voltammetry at the range of working potentials of various biochemical molecules in interstitial fluid.

## 1. Introduction

The desire to lead a healthy life and increased life expectancy are gradually changing the medical service paradigm from ‘diagnosis and treatment’ to prevention and management [1,2,3]. To take advantage of this trend, user-customized healthcare technologies that aid lifestyle management, such as the management of food intakes, weight, and body shape, have received much attention [4,5]. To realize such healthcare technologies, sensors are required that are capable of accurately monitoring user biometric variables, conveniently yet inexpensively. Several multifunctional sensors have been shown to extract different types of biometric information from trace amounts of blood components [6,7,8] and to detect vital sign signals generated by organs, such as electromyographic and electrocardiographic signals [9,10]. Of the various sensors developed, microneedle-based sensors have been used in wearable healthcare devices owing to their advantages of minimal invasiveness and user-friendliness.

Microneedle technology was initially introduced as a new drug delivery method for drugs, vaccines, and cosmetics to overcome the physical and chemical limitations posed by the stratum corneum [11,12,13]. By mounting a three-electrode system consisting of working, counter, and reference electrodes, microneedles have evolved into a sensor technology to monitor the levels of biometrically valuable biochemicals in biofluids using electrochemical detection methods [14,15], such as cyclic voltammetry and chronoamperometry. Unlike subcutaneous vein detection using hypodermic needles, the microneedle sensor penetrates the skin minimally and controllably to a depth of several hundred micrometers, which provides a patient-compliant and painless way of obtaining biometric data from interstitial fluid. The ability of the sensor to continuously acquire biometric information in real time is also considered important for its practical implementation. The detection of various analytes, such as glucose [16], L-dopa [3], alcohol [2], and uric acid [17,18], in interstitial fluid under human skin using microneedle sensors has actually been previously demonstrated.

To reduce skin inflammation, as well as to eliminate electrical background noise in electrochemical sensing, a polymeric material, having biocompatibility and biodegradability, is used as a material for microneedles. Representative materials include PLA [19,20], polyurethane (PU) [21], poly(ethylene glycol) (PEG) [22], polystyrene (PS) [23], and poly(methyl methacrylate) (PMMA) [24,25]. For the manufacturing of microneedles, they could be produced directly from the polymeric materials using methods of melt-drawing [26], droplet air blowing [27], and 3D printing [28]. Meanwhile, polymer casting processes, such as high-temperature embossing [29,30], injection molding [31], and solution casting [32,33], have been widely used to fabricate large numbers of microneedles uniformly and productively. As for a mold material of polymer castings, a silicone-based elastic polymer, such as polydimethylsiloxane, was typically used [29,32,34,35], which facilitates the molding and detachment of high-aspect-ratio polymer microneedles from the mold owing to its low surface energy. However, the elastic mold has a disadvantage in that it is easily deformed by the temperature and pressure applied to the polymer casting, which causes difficulties in reproducing the shape of the microneedle. In particular, in manufacturing a microneedle sensor that has to go through several subsequent processes, such as metal deposition and coating of a sensing material, the mold deformation is a significant cause of lowering production yield.

In this work, we present a fabrication procedure suitable for the mass production of microneedle sensors, which can be utilized for the electrochemical detection of various biomarkers in biofluids. Microneedle sensors were fabricated by thermal compression molding of PLA into a laser-machined PTFE mold. Working, counter, and reference electrodes were formed on sensor surfaces by Au sputtering through a stencil mask. The operational stabilities of the fabricated sensor were confirmed by linear sweep voltammetry (LSV) and cyclic voltammetry (CV) using a range of working potentials targeting various biochemical molecules in interstitial fluid.

## 2. Materials and Methods

### 2.1. Materials

A roll of 1.75 mm diameter PLA filament was purchased from Sondori, South Korea, and cut into lengths of ≤1 cm with scissors for thermocompression molding. A sheet of PTFE (3 mm thick) was purchased from Mirae International Trading, Gunpo, South Korea. The release agent (Easy-Lease™) was used to enable PLA microneedles to be detached from PTFE molds after thermocompression molding and was purchased from Easy Composites Ltd., Longton, UK. Adhesive film (Tegaderm™ transparent film dressing), which was used to attach the microneedle sensor to skin, was purchased from 3M, South Korea, and phosphate buffered saline (PBS 1X) was from Lonza, Switzerland.

### 2.2. Measurements and Instrumental 

A computer-aided engraving machine equipped with a CO_2_ laser (KL-900L, Woosung E&I Co., Pyeongtaek, South Korea) was used to fabricate PTFE molds. This machine can process an area of 1200 × 900 mm^2^ with a scan resolution of 2500 dots per inch (DPI) and a positional accuracy of 10 μm, and has a laser power of up to 100 W. To process PTFE, the engraving depth was controlled by adjusting laser movement during irradiation at a fixed duty cycle of 50%. The working distance between the laser beam source and the PTFE was fixed at 1 cm. Scanning electron microscopy (CX-200TM, COXEM, Daejeon, South Korea) was used at an acceleration voltage of 10–15 KeV to observe the morphologies of the microneedles produced. Electrodes were formed by sputtering Au on sensors through a stainless steel (SUS) stencil mask using a metal sputtering unit (Q300T D Plus, Quorum, Laughton, UK) at a current of 100 mA for 420 s. Linear sweep voltammetry (LSV) and cyclic voltammetry (CV) measurements were performed using a computer-controlled voltage meter (CS310, Corrtest Instruments, Wuhan, China) with a potential resolution of 10 μV. Operation stabilities of sensors were assessed using LSV and CV measurements, which were performed in a PBS solution at a scan rate of 5 mV/s over the potential sweep range of −1.0 to +1.0 V and +0.1 to +0.6 V, respectively, versus an Ag/AgCl reference electrode.

## 3. Results and Discussion

### Laser Machining of the PTFE Mold

**Figure 1:**
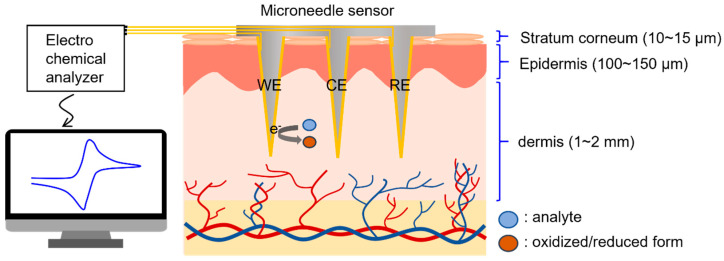
Illustration of the electrochemical detection of biomarkers in dermal interstitial fluid.

A conceptual diagram of the electrochemical detection of biomolecules in interstitial fluid using the microneedle sensor is provided in Figure 1, which shows microneedles penetrating the epidermis and accessing interstitial fluid. This fluid is representative of the fluid between cells and blood vessels and accounts for 70% of dermis by volume [36]. The composition of interstitial fluid is similar to that of blood plasma [37,38], except for high molecular weight proteins, because equilibrium between plasma and interstitial fluid is achieved by capillary walls, which allow biomolecules with molecular weights of ≤10,000 Da to pass freely.

The microneedle sensors were designed to diagnose and monitor health by accessing interstitial fluid under the epidermis in a minimally invasive manner. To meet this design objective, a method of manufacturing biocompatible microneedles several hundred micrometers or more long with a high aspect ratio and mechanically robust enough to withstand forces during skin insertion was required.

To ensure the manufacturing process allowed straightforward control of microneedle length, we produced sensors by the thermal compression molding of PLA into laser-engraved PTFE molds. We selected PLA for this purpose having considered other candidate biocompatible materials used to produce microneedles, such as polyurethane, polyethylene, polystyrene, and poly(methyl methacrylate), because PLA is an FDA-approved generally recognized as safe (GRAS) polymer with excellent mechanical properties and electrochemical stability. The PTFE mold engraving depth, which determined the needle length after PLA molding, was adjusted by modulating the laser scan speed; other laser process parameters, such as power, duty cycle, and working distance, were fixed. A detailed description of the conditions used for laser engraving is provided in the Measurements and Instrumental section above.

**Figure 2:**
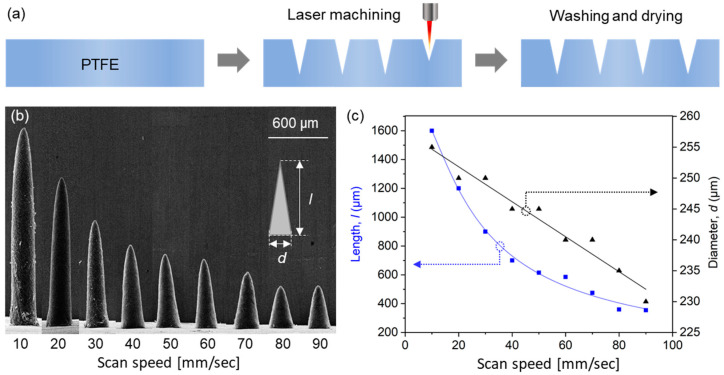
(**a**) Fabrication process used to produce PTFE molds by laser engraving. (**b**) SEM images of the PLA microneedles produced using molds laser machined at different scan speeds. (**c**) Lengths and diameters of fabricated microneedles.

A schematic of the laser engraving of PTFE molds is provided in Figure 2a. A PTFE film was engraved with a negative of microneedle shapes using a CO_2_ laser. The small amount of debris generated during laser processing was removed by washing molds with acetone in an ultrasonic bath and drying at room temperature. To evaluate engraving depths, we measured the lengths of microneedles produced using molds that had been engraved using different laser scan speeds.

Scanning electron microscopy (SEM) images of PLA microneedles fabricated at different laser scan speeds are shown in Figure 2b. The engraving depth increased as scan speed decreased, which was attributed to the time the laser beam impacted the PTFE surface. Microneedle lengths and diameters (defined as diameters at microneedle bases, as shown in the inset of Figure 2b) were determined by SEM. Figure 2c shows the results of measuring the average length and diameter of 10 or more microneedle specimens. As shown in the figure, microneedle lengths were adjustable from 390 to 1600 μm using scan speeds of 90 and 10 mm/s, respectively, at which needle diameters increased slightly from 232 to 255 μm, respectively, that is, they were smaller than the outer diameter of a 31-gauge syringe needle (261 μm). Given a depth from the stratum corneum to dermis of <200 μm (Figure 1) and the insertion depth to minimize the pain caused by needle insertion [39], we chose to use a needle length of 600 μm, which corresponded to a scan speed of 40 mm/s.

**Figure 3:**
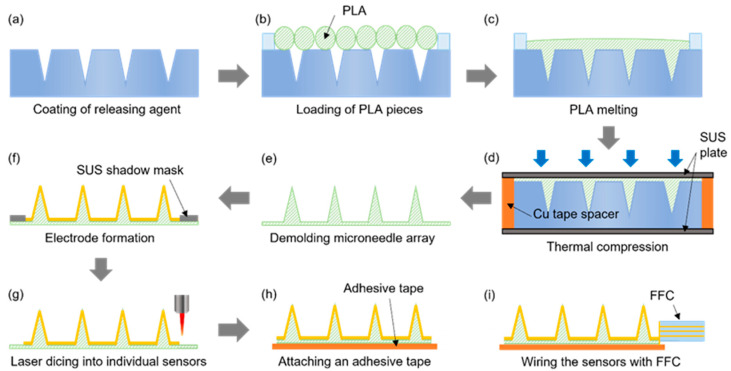
The procedure used to fabricate microneedle sensors. (**a**) Coating of releasing agent. (**b**) Loading of PLA pieces. (**c**) PLA melting. (**d**) Thermal compression. (**e**) Demolding microneedle array. (**f**) Electrode formation. (**g**) Laser dicing into individual sensors. (**h**) Attaching an adhesive tape. (**i**) Wiring the sensors with FFC.

The microneedle sensor manufacturing procedure, which is schematically provided in Figure 3, was designed to be suitable for the mass production of multiple microneedle sensors on a 4-inch wafer. Initially, the laser-processed PTFE mold was coated with a mold release agent to facilitate the detachment of PLA microneedles (Figure 3a) and then dried under ambient conditions. PLA filaments were cut into ≤1 cm lengths and placed on the PTFE mold (Figure 3b), and then heat-treated in a vacuum oven for 30 min at 200 °C, which is slightly higher than the melting point of PLA (~180 °C). Thus, the heat treatment melts the PLA, which then fills the mold under vacuum conditions (Figure 3c). Thermal compression molding was then performed using a press at 220 °C and 1.0 MPa for 5 min to control the thickness of the microneedle sensor substrate and ensure the accuracy of the molding procedure (Figure 3d). To ensure a uniform temperature distribution during thermal compression, the specimen was sandwiched between two stainless steel (SUS) plates. A copper tape spacer was attached around the edges of the bottom SUS plate to produce sensors with a substrate thickness of ~200 μm. After thermal compression, the substrate with PLA microneedles was detached from the PTFE mold (Figure 3e). Subsequently, the microneedle electrodes on each sensor were separated into working, counter, and reference electrode regions by sputtering the PLA microneedle substrate with a 200 nm thick Au film through a SUS stencil mask (Figure 3f). The multiple microneedle sensors fabricated on a 4-inch diameter wafer were then separated into individual sensors by laser dicing (Figure 3g). An adhesive film was then attached to the back of each sensor to allow application to skin, and finally, a flexible flat cable (FFC) was connected to complete the sensor fabrication (Figure 3h,i).

**Figure 4:**
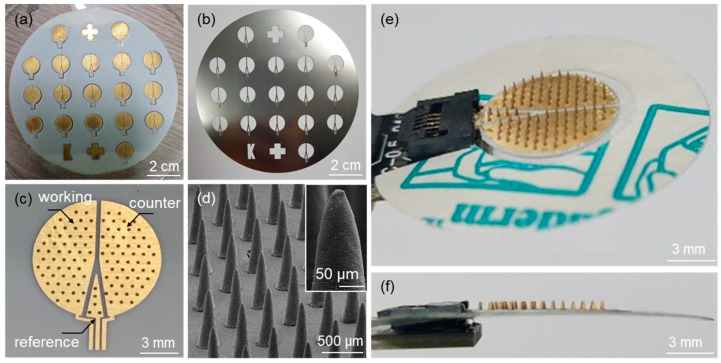
Optical images of (**a**) microneedle sensors fabricated on a 4-inch wafer size after the formation of Au electrodes, (**b**) the SUS stencil mask used for the Au sputtering process, and (**c**) the microneedle sensor separated from the substrate by laser dicing. (**d**) SEM image of an array of microneedles on the sensor. Optical images of (**e**) top and (**f**) side views of the microneedle sensor equipped with an adhesive film and the FCC connection.

Figure 4a shows an optical image of the fabricated microneedle sensors on a 4-inch wafer after the deposition of Au electrodes using the SUS stencil mask (Figure 4b). An optical image of a microneedle sensor separated from the substrate by laser dicing is shown in Figure 4c. The figure also shows the electrode area divided into working, counter, and reference electrodes. SEM confirmed that an array of microneedles on the sensor was successfully fabricated (Figure 4d). Figure 4e,f show top and side view images of a microneedle sensor equipped with an adhesive film for skin attachment and an FCC connection, respectively.

**Figure 5:**
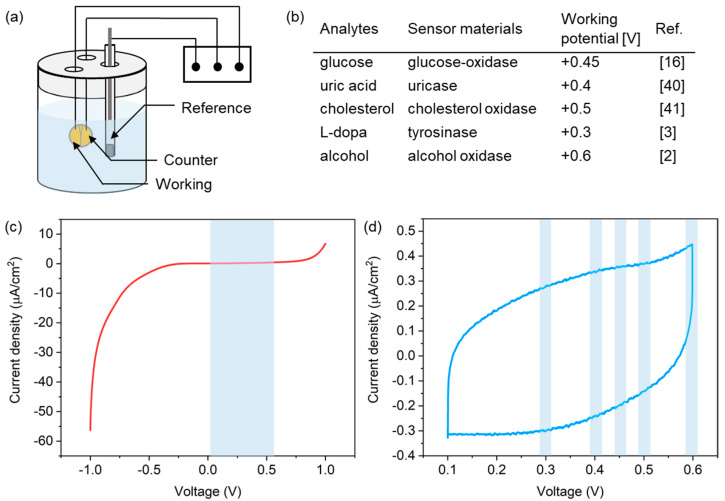
(**a**) Experimental setup used for LSV and CV measurements. (**b**) Working potentials of the redox reactions of several analytes. Current responses from the microneedle sensor in PBS solution during (**c**) LSV and (**d**) CV measurements in PBS.

To successfully achieve electrochemical detection of biomarkers in biofluids, the PLA microneedle sensor preferentially requires stable operation in a buffer solution for supporting biochemical molecules. To validate the operational stability of the microneedle sensor, we checked for the presence of any redox peaks possibly caused by salts in the buffer solution by linear sweep voltammetry (LSV) and cyclic voltammetry (CV), as shown in the experimental setup in Figure 5a. A solution of phosphate buffered saline (PBS 1X, pH 7.4), which is an isotonic solution commonly used in biological research studies, was used for the tests. The scan rate was fixed at 5 mV/s and a commercial Ag/AgCl reference electrode was used. Considering the range of working potentials of various biochemical molecules (Figure 5b) [2,3,16,40,41], measurements were performed over the potential range of −1.0 to +1.0 V and +0.1 to +0.6 V, respectively, versus the Ag/AgCl reference electrode.

Figure 5c shows LSV current responses observed, which indicated the absence of redox reactions in the potential range between 0.1 V and 0.6 V (indicated by the blue region in the figure), which covered all working potentials of the analytes shown in Figure 5b. A CV curve is shown in Figure 5d, and no current peak was detected at the working potentials of the redox reactions of analytes. These results indicate that the PLA microneedle sensor presented here operated stably for the detection of biochemicals.

## 4. Conclusions

We fabricated PLA microneedle sensors by thermocompression molding of PLA into a PTFE mold engraved by CO_2_ laser machining. Microneedle geometry was controlled by modulating the laser scan speed. Microneedle lengths of 390 to 1600 μm were achieved by reducing scan speeds from 90 to 10 mm/s, respectively, while microneedle diameters increased slightly from 232 to 255 μm. Sensor fabrication was completed by forming working, counter, and reference electrodes on the sensor, which was accomplished by Au sputtering through an SUS stencil mask. The presented fabrication process was found to be highly effective at producing microneedles with high aspect ratios and various lengths reproducibly. Furthermore, the entire process was designed to be suitable for the mass production of multiple microneedle sensors on 4-inch wafers. Finally, the operation stability of the fabricated microneedle sensors was confirmed using LSV and CV measurements performed at the working potentials of various biochemical molecules in interstitial fluid. We believed that the PLA microneedle sensors presented here are capable of providing an effective sensing platform for the detection of biochemicals of interest.

## Data Availability

Data are contained within the article.
